# HPLC-DAD Determination of Nitrite and Nitrate in Human Saliva Utilizing a Phosphatidylcholine Column

**DOI:** 10.3390/molecules24091754

**Published:** 2019-05-06

**Authors:** Małgorzata Tatarczak-Michalewska, Jolanta Flieger, Justyna Kawka, Wojciech Płaziński, Wojciech Flieger, Eliza Blicharska, Dariusz Majerek

**Affiliations:** 1 Department of Analytical Chemistry, Medical University of Lublin, Chodźki 4A, 20-093 Lublin, Poland; justyna.kawka@umlub.pl (J.K.); eliza.blicharska@umlub.pl (E.B.); 2 Jerzy Haber Institute of Catalysis and Surface Chemistry, Polish Academy of Sciences, Niezapominajek 8, 30-239 Krakow, Poland; wojtek_plazinski@o2.pl; 3 II Faculty of Medicine with English Language Division, Medical University of Lublin, Aleje Racławickie 1, 20-059 Lublin, Poland; wwoj24@wp.pl; 4 Department of Applied Mathematics, University of Technology, Nadbystrzycka 38D, 20-618 Lublin, Poland; majerek@gmail.com

**Keywords:** phosphatidylcholine column, nitrite, nitrate, human saliva, sustainable chemistry

## Abstract

The aim of this research was to optimize the separation and quantitative determination of nitrites and nitrates in human saliva. HPLC with UV absorption (HPLC/DAD) using a phosphatidylcholine column (IAM.PC.DD2 Regis HPLC) was applied in this assay. Nitrates were detected directly by their absorbance at 210 nm, whereas nitrites were detected after oxidation to nitrates by potassium permanganate at acidic conditions. The kinetics of the permanganate–nitrite reaction was measured chromatographically. The calibration graph for nitrates was linear in the range of 0.5–35 µg mL^−1^ with a correlation coefficient of 0.9999. The limit of detection was 4.56 ng mL^−1^. The calibration graph for nitrites (after oxidation to nitrates) was linear in the range of 0.5–15 µg mL^−1^ with a correlation coefficient of 0.9972. The limit of detection was 4.21 ng mL^−1^. The nitrate concentrations in the saliva samples were found in the range of 8.98–18.52 μg mL^−1^, whereas nitrite was in the range of 3.50–5.34 μg mL^−1^.

## 1. Introduction

Nitrate (NO_3_^−^) and nitrites (NO_2_^−^) are interconvertible as the components of the nitrogen cycle in the natural environment as well as the human body via the nitrate–nitrite–nitric oxide pathway [1].

The endogenous source of these ions is the l-arginine–NO pathway, whereas the main dietary source is green leafy vegetables such as celery, lettuce or spinach, accounting for almost 90% of daily nitrate intake [2,3]. Nitrate is also found in drinking water, particularly in agricultural areas, as it is used in fertilizers. In turn, nitrite is used commonly as an additive in meat to improve its color and taste as well as to prevent the growth of *Clostridium botulinus* [4,5,6]. 

Nitrates are biologically inert but could be hazardous after conversion to nitrites. The reduction process is able to proceed in vivo. Salivary bacterial reduction has been recognized as a dominant metabolic conversion process, as a substantial amount of ingested nitrate is concentrated in saliva [7]. Several enteric bacteria such as *Escherichia coli*, *Lactobacillus* and *Bifidobacterium* species have also shown the ability for the catalytic reduction of nitrate, but the importance of this pathway has been little studied [8,9].

Nitrites, by the conversion of hemoglobin to methemoglobin, are responsible for methemoglobinemia, which is particularly hazardous for infants and pregnant woman. Moreover, nitrites are able to react with secondary amines and amides to form carcinogenic *N*-nitrosamines in the stomach [10,11,12,13].

The nitrate/nitrite levels depend on dietary contributions, but it was proven that, in diseases with endothelial dysfunction, these levels are lower [14], whereas regular exercise or systemic inflammatory disorders result in higher levels of both [3]. 

To date, anion exchange and ion-pair chromatography have been applied for the determination of nitrite and nitrate anions. Both methods utilize spectrophotometry, electrochemistry, chemiluminescence or fluorescence as their detection modes [15,16,17,18,19,20,21,22,23,24,25].

Nitrites and nitrates can be measured either directly by their conductivity or absorbance in the range of 210–220 nm. The most common methodology involves the evaluation of nitrates by reducing them to nitrites on a Cu/Cd column and further derivatization leading to a finally quantified fluorescent, luminescent or long-wavelength-absorbing product of the Griess reaction of nitrite with sulfanilamide and *N*-(1-naphthyl)ethylenediamine. In turn, after the reaction of nitrite with 2,3-diaminonaphthalene (DAN), a highly fluorescent compound can be created [15,25]. It should be emphasized that the post- and pre-column derivatizations are characterized by poor sensitivity and specificity [26]. Apart from that, the high level of chlorides causes interferences in direct nitrite/nitrate detection. Therefore, the isolation of target analytes by clean-up procedures eliminating interfering compounds is frequently required before analysis. He et al. [21] proposed an ionic liquid dispersive liquid–liquid microextraction (IL-DLLME) of nitrite ions followed by ion-chromatography utilizing a column with a strong anion exchanger packing material, such as polymethacrylate resin with a quaternary ammonium functional group. Ion-pair (IP) chromatography is more accurate, sensitive, and selective. Ferreira and Silva proposed the chromatographic separation of nitrite and nitrate in ham samples using a HyPurity C18 column and gradient elution with 0.01 M n-octylamine and 5 mM tetrabutylammonium hydrogen sulfate [22]. 

In 2003, Hu et al. developed a phosphatidylcholine-bonded packing material to separate the inorganic anions [27]. This new type of biomimetic stationary phase has been created by incorporating liposomes constructed from dimyristoyl phosphatidylcholine (DMPC) onto RP-columns. They noticed that the DMPC stationary phase recognizes mono- and divalent anions in a quite distinct manner in comparison to anion-exchange packing materials. Currently, stationary phases with phospholipids are being prepared commercially by the covalent binding of single phospholipids or their analogues to silica propyl-amino particles [28,29]. 

The objective of this study was to optimize the conditions of separation and quantitative determination of nitrites and nitrates in human saliva using chromatography on a commercially available phosphatidylcholine column (IAM.PC.DD2 Regis HPLC). In this assay, nitrates have been determined directly by their absorbance around 210 nm, whereas nitrites were determined after being oxidized to nitrate by potassium permanganate at acidic conditions. The method proposed in this study has not been described in the literature so far. The work presents new possibilities of using the IAM column. The process of the deproteinization of the samples using high-temperature and chromatographic analysis carried out without the use of organic solvents meet the criteria of green chemistry because it minimizes the use and generation of hazardous substances in the environment.

## 2. Results and Discussion

### 2.1. Optimization of Nitrate/Nitrite Separation

**Figure 1:**
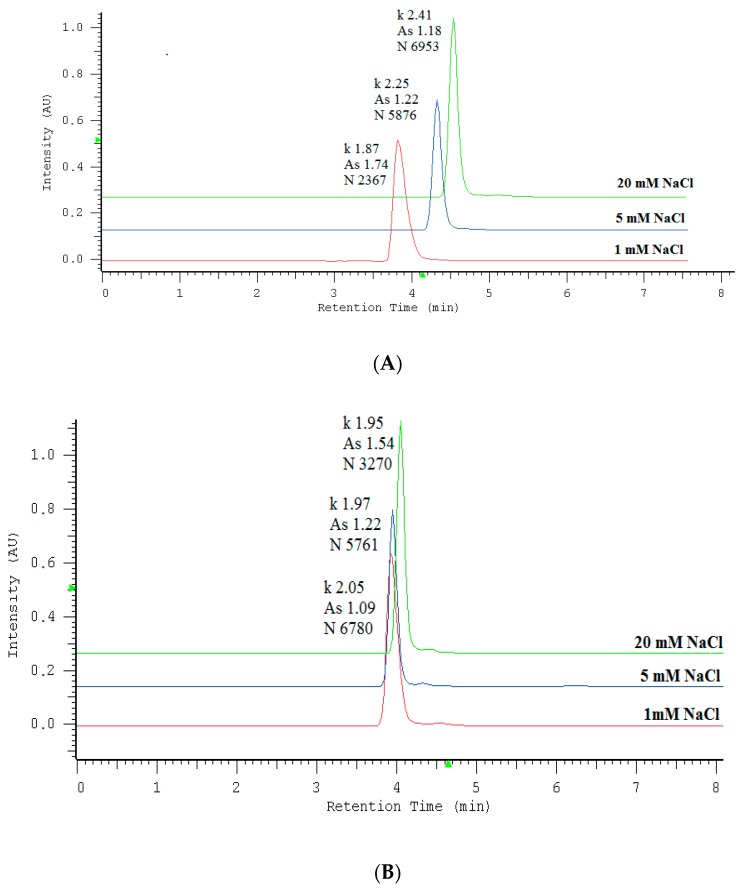
Chromatograms illustrating influence of NaCl in the mobile phase on the parameters of NO_3_^−^ (**A**) and NO_2_^−^ (**B**) peaks. The column was IAM.PC.DD2 Regis HPLC Agilent Technologies, the flow rate of the mobile phase was 0.5 mL/min, the injected volume 3 μL, and the analytical wavelength of 210 nm. The abbreviation *k* stands for retention factor, *A*_s_ denotes the symmetry factor and *N* is the theoretical plate number.

**Figure 2:**
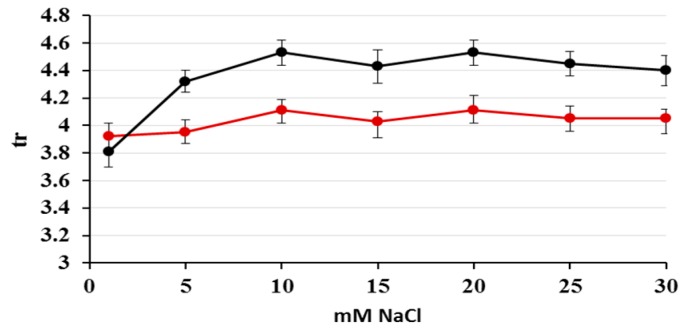

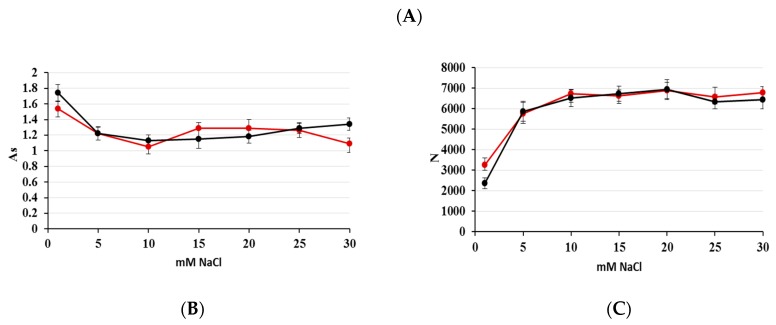
The effect of NaCl concentrations in the range of 1–30 mM (1, 5, 10, 15, 20, 25, 30 mM) in the aqueous mobile phase on NO_2_^−^ (red line) and NO_3_^−^ (black line) peak parameters: tr (**A**), *A_s_* (**B**), *N* (**C**). Abbreviations: tr, *A_s_* and *N* denote retention time in minutes, asymmetry factor (the Hitachi High-Performance Liquid Chromatography System Manager HSM software uses the following equation to calculate asymmetry: *A_s_* = 1/2(1 + B/A), where A and B are evaluated at a 5% peak height of an appropriate peak), and the number of theoretical plates (*N* values were calculated according to The United States Pharmacopeia (USP): *N* = 16(tr/W)^2^, where W equals the peak width obtained by drawing tangents to each side of the peak and calculating the distance between the two points where the tangents meet the baseline), respectively.

Aqueous solutions of sodium salts of UV-absorbing anions of nitrates and nitrites were analyzed using commercially available phosphatidylcholine stationary phase IAM.PC.DD2 Regis and aqueous NaCl as the mobile phase. The proposed system showed the ability to separate these anions within 5 minutes. The effect of NaCl concentrations in the range of 1–30 mM (1, 5, 10, 15, 20, 25, 30 mM) in the aqueous mobile phase on retention (*k*), peak symmetry (*A_s_*) and the theoretical plate number (*N*) for NO_2_^−^ and NO_3_^−^ was studied. Appropriate chromatograms are presented in Figure 1A,B and curves in Figure 2A–C. 

Plots of the retention time versus concentration of NaCl (Figure 2A) show that the retention for the NO_3_^−^ ions increased slightly as NaCl content increased, while that for the NO_2_^−^ ions remained almost constant. The addition of sodium chloride to the mobile phase caused the improvement of the peak symmetry and the efficiency of the system, expressed as the theoretical plate number. The most significant changes of peak parameters occurred in the range of 0–10 mM NaCl in the mobile phase. As can be observed, the mobile phase above 10 mM NaCl ensured constant retention, satisfactory symmetry in the range of *A_s_* from 0.8 to 1.2, and efficiency above 6000 *N* for both peaks. For further experiments, 20 mM NaCl was chosen as the mobile phase. At the above conditions, the retention time of nitrite was 4.11 min, whereas for nitrate this was 4.53 min. It has been previously established that anions are retained on the basis of a solvation-dependent mechanism among other factors. Performed optimization confirmed that nitrates possessing smaller free energies of hydration (ΔG_hyd_ = −300 kJ mol^−1^) exhibited longer retention, whereas nitrites with larger free energies of hydration (ΔG_hyd_ = −330 kJ mol^−1^) showed weaker retention [30]. Considering the presence of the quaternary ammonium groups with positive electrostatic potential (N^+^) on the surface of the stationary phase, an anion-exchange mechanism can also contribute to anion retention. Both mechanisms are affected by the eluent’s ionic strength, which is the reason behind the fact that if NaCl concentration increases, all peak parameters and the system selectivity undergo visible improvement. In accordance with the location of chloride ions in the Hofmesiter series [31] reflecting the hydration free energies of ions, they are hydrated more strongly compared to nitrate/nitrite anions. This preference of chloride ions to their strong interactions with water-based hydrogen, which can penetrate the ionic van der Waals shell, decreases the hydration of investigated anions undergoing stronger retention. Recalling chromatography using chaotropic additives to the eluent [32,33], we are dealing here with the reverse effect; i.e., the influence of kosmotropic chloride ions on the separation of chaotropic analytes.

### 2.2. Quantification of Nitrate/Nitrite by Oxidation Using Potassium Permanganate

**Figure 3:**
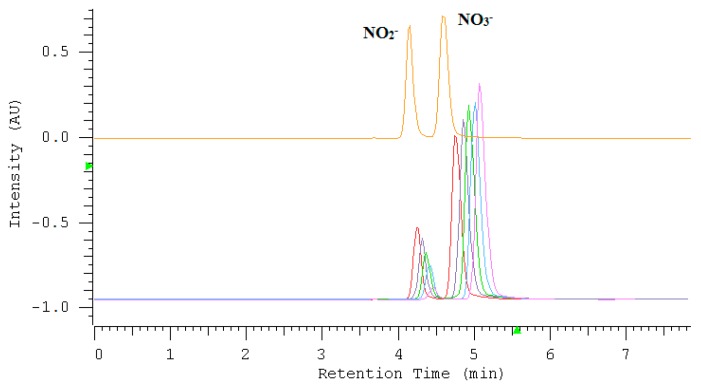
The overlapped chromatograms illustrating the peaks of nitrate/nitrite after oxidation reaction using potassium permanganate under acidic conditions. The upper chromatogram illustrates the separation of a mixture containing nitrate and nitrite for the identification of peaks.

In this research, nitrates have been determined directly by their absorbance at 210 nm, whereas nitrites were determined after oxidizing to nitrate by potassium permanganate under acidic conditions. In order to optimize the composition of the mixture, a solution containing 4 mL of 100 ppm NO_3_^−^, 4 mL of 100 ppm NO_2_^−^, 10 μL HClO_4_ and 0.02 M KMnO_4_ (50, 80, 100, 120, 150 and 170 μL) was prepared and then made up to 10 mL with deionized water. As a result of the addition of further portions of KMnO_4_, the nitrite peak decreased in height, whereas the nitrate peak demonstrated the opposite behavior. The subsequent stages of the analysis are visualized by the chromatograms shown in Figure 3. 

**Figure 4:**
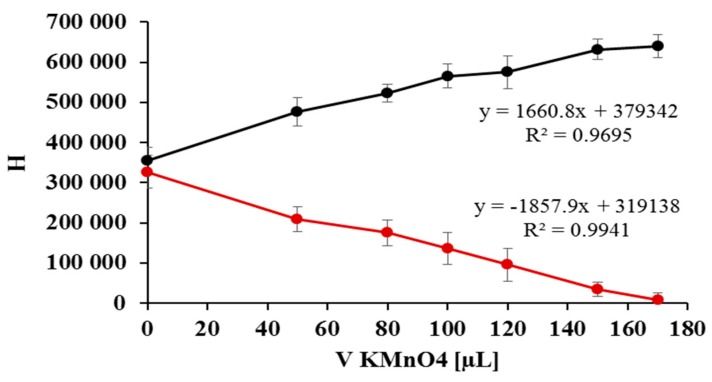
Linear dependencies of the peak height for nitrate (black line)/nitrite (red line) versus the volume of KMNO_4_.

The gradual decrease in the height of the nitrite peak corresponds directly to the increase in the height of the nitrate peak. The linear dependencies of the peak height versus the volume of KMnO_4_ solution for nitrates/nitrites are in a relation to each other similar to mirror reflections. This is confirmed by the values of the slopes, which have similar values but opposite signs (Figure 4).

### 2.3. The Kinetic Study of the Permanganate–Nitrite Reaction in Acidic Media

To investigate the kinetics of the reaction, several solutions were prepared—a solution containing 0.5 mL of a solution of NO_3_^−^ at a concentration of 100 ppm, and a solution of 0.5 mL of NO_2_^−^ at a concentration of 100 ppm, 10 μL HClO_4_ and 25 μL of 0.02 M KMnO_4_ (to obtain a persistent pale pink coloration of the solution)—and then the solution was made up to 10 mL with deionized water. The following redox reaction was studied:

2MnO_4_^−^ + 5NO_2_^−^ + 6H^+^ ➞ 2Mn^2+^ + 5NO_3_^−^ +3H_2_O

The concentration of KMnO_4_ as well as the concentration of hydrogen ions in the sample were constant at 0.05 mmol L^−1^ and 11.46 mmol L^−1^, respectively. Since it has been proven by Dózsa and Beck [34] that the rate of the reaction is independent of the permanganate concentration in the range of 5 × 10^−5^–5 × 10^−3^ M, and between pH 1.8 and 2.6, the rate of this reaction can be described by the following equation:

Rate = *k*_l_[NO_2_^−^] + *k*_2_[NO_2_^−^]^2^ + *k*_3_[NO_2_^−^]^3^[H^+^]^3^ (1)

where *k*_l_, *k*_2_ and *k*_3_ are the rate constants representing the particular rate-determining steps. The detailed description of these steps is given in [34], as well as the derivation of the corresponding rate equation. 

Although the rate constants are provided by the authors of the original paper [34], the inherent differences between the systems (e.g., chemical composition, ionic strength, analytical setup, etc.) require performing independent calculations. Instead of directly adopting the rate constant values determined in [34], we used only the mathematical form of the corresponding model and determined the reaction rate constants separately, according to the criterion of the agreement with the measured experimental data. Such a procedure represents some degree of flexibility that reflects the possible differences between the real experimental systems. This has been done by numerically solving the corresponding partial differential equation with the initial conditions accounting for the actual reagent concentrations. The solving procedure was performed iteratively, by accounting for various sets of *k*_l_, *k*_2_ and *k*_3_ values, and the criterion of acceptance of the final values was the minimization of the total deviation from the experimental concentrations collected during the experimental part of the study. This was achieved by the hand-written code and built-in subroutine (*NDSolve*) in the Mathematica 8.0 software. 

**Figure 5:**
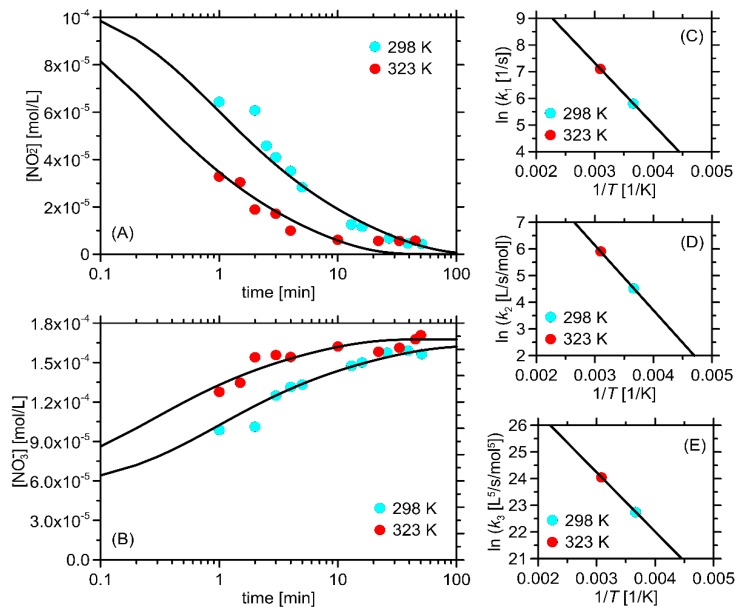
The dependences of (**A**) the decreasing concentration of NO_3_^−^ over time, (**B**) the increasing concentration of NO_2_^−^ over time. The logarithmic scale was applied for clarity. (**C**,**D**,**E**) The Arrhenius plots (i.e., the dependence of the logarithm of the rate constants (k_1–3_) on the reciprocal of the absolute temperature) for the three reaction stages. The initial concentration of NO_2_^−^ is equal to 0.11 mmol L^−1^, whereas that of NO_3_^−^ equals 0.16 mmol L^−1^. The concentrations of KMnO_4_ and H^+^ are constant and equal to 0.05 mmol L^−1^ and 11.46 mmol L^−1^, respectively. The points correspond to the experimental data, whereas the solid lines correspond to the predictions of the corresponding rate equation (proposed in [34]).

For the rate constants, the following values were obtained at 25° C: *k*_l_ = 3.3 × 10^−2^ s^−1^, *k*_2_ = 92 M^−1^ s^−1^, *k*_3_ = 7.7 × 10^−9^ (M^−5^ s)^−1^. When the temperature increased up to 50 °C, the constants also increased to the following values: *k*_1_ = 1.2 × 10^−3^ s ^−1^, *k*_2_ = 366 M^−1^ s^−1^, *k*_3_ = 2.72 × 10^−10^ (M^−5^ s)^−1^. The availability of the data measured for the two different temperatures allowed the application of the Arrhenius relationship (Figure 5) and the calculation of the corresponding thermodynamic parameters: namely, the enthalpies of activation accompanying the particular reaction stages are equal to ΔH_1_ = 19.2 kJ/mol, ΔH_2_ = 20.2 kJ/mol and ΔH_3_ = 18.5 kJ/mol. It may be seen that the obtained rate constant values are consistent with those measured spectrophotometrically in the independent study [35], which additionally confirms the correctness of the assumed reaction mechanism. 

### 2.4. Linearity and Sensitivity

The quantification of nitrates was based on an external standard method utilizing the calibration relationship between peak heights vs. the concentration of nitrate standards, whereas the quantification of nitrites was based on the correlation obtained between the increase of the nitrate peak height after oxidation versus nitrite ion concentration. All calibration parameters are summarized in Table 1. The calibration curves of eight concentrations showed a good linear response for both analytes in the range of 0.5–35 µg mL^−1^ for nitrate and 0.5–15 µg mL^−1^ for nitrite. The detection limits were 4.56 ng mL^−1^ and 4.21 ng mL^−1^ for nitrate and nitrite, respectively. 

### 2.5. Recovery Study

The relative recovery values were estimated by measuring samples of saliva spiked with nitrite and nitrate at four concentrations with 6 replicates of each. Nitrates were measured before the oxidation step, whereas nitrites were measured after oxidation as an increase of the nitrate peak height. The blank saliva sample was measured simultaneously. The determined peak height values before the oxidation step were substituted into the following equation, [(after spiking − before spiking)/added] × 100, to calculate the percentage extraction yield for nitrate, whereas the nitrite content was measured after the oxidation step substituting an increase of the nitrate peak height (Δ = nitrate peak height after oxidation − nitrate peak height before oxidation) into the following equation: [(Δ_after spiking_ − Δ_before spiking_)/added] × 100%. No statistically significant differences were noticed for the lower and higher concentrations, as shown in Table 2. As shown, the recovery values of nitrate and nitrite varied between 93.33–98.65%, and 86.90–104.23%, respectively.

The method provided satisfactory precision expressed by relative standard deviation values. Precision values expressed as relative standard deviation (RSD)% values were lower than 4% for repeatability (intra-day precision) and lower than 6% for intermediate precision (inter-day precision).

### 2.6. Stability Tests

The standards at three concentration levels (5, 12.5 and 25 ppm) were heated at 60, 70, 80, 90, and 100 °C for 15 min before IAM-HPLC-DAD analysis. Secondly, the time of heating at 100 °C was studied for 5, 15, 35, 45 and 60 min. 

**Figure 6:**
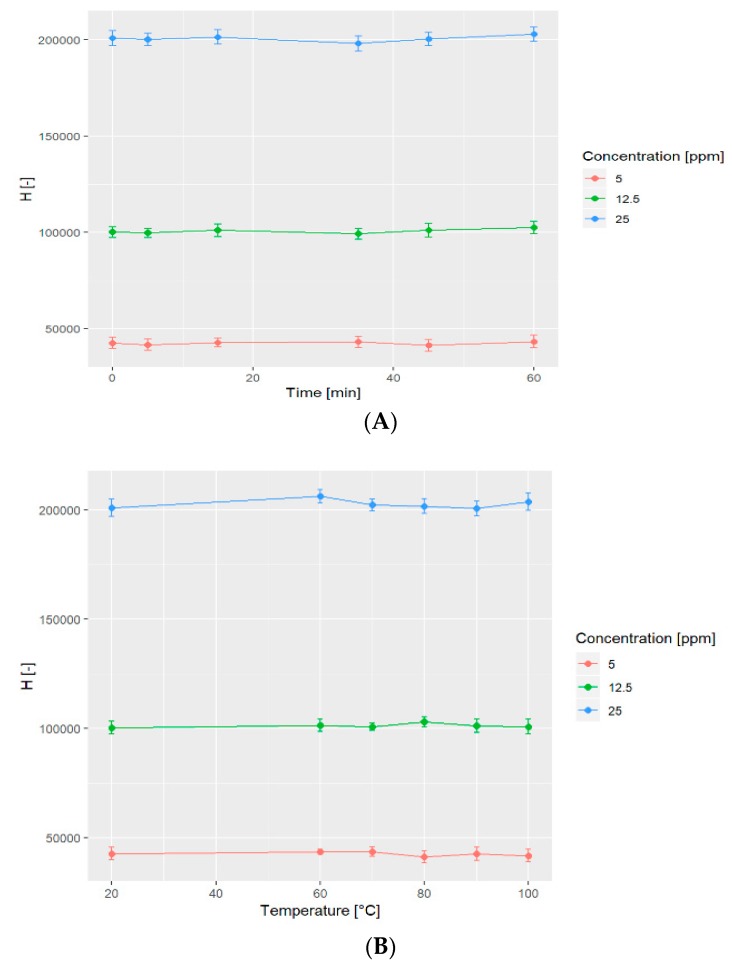
Influence of time of heating at 100 °C (**A**) and temperature of sample heating for 15 min; (**B**) the height (H) of the nitrate peak.

The aim of the statistical analysis was to find if there is an influence of time and temperature on the height of peaks and also the concentration of nitrate. In order to answer these questions, we conducted an appropriate ANOVA test. All the assumptions of analysis of variance were checked and fulfilled. The test was conducted on each concentration level separately. All hypotheses were not rejected, based on *p*-values greater than 0.05. For the temperature of heating, *p*-values were as follows from the lowest to the highest concentration: *p* = 0.76, *p* = 0.8, and *p* = 0.26. For the time of heating at 100 °C, *p*-values were, respectively, *p* = 0.92, *p* = 0.72, and *p* = 0.62. The obtained data means that there is no significant influence of time and temperature on the height of peaks of nitrate. The average peak height at each concentration level was stable, as shown graphically in Figure 6.

The peak symmetry, reproducibility of retention time and the peak height of nitrate were evaluated in samples of saliva and the water system without and after oxidation reaction. The results presented in Table 3 showed that the stability and reproducibility of the presented method were in an acceptable range.

### 2.7. Application of the Assay

**Figure 7:**
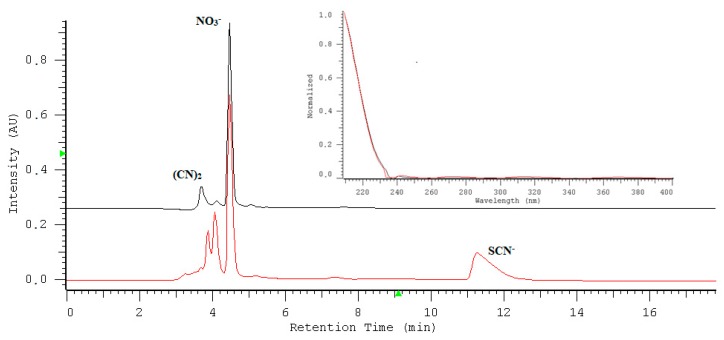
The overlapped chromatograms; the red line represents the separation of the saliva sample before oxidation, and the black line that after oxidation. Insert: UV-absorption spectra of nitrate in water (the back line) and in the saliva sample (the red line) recorded in the range of 190 to 400 nm.

**Figure 8:**
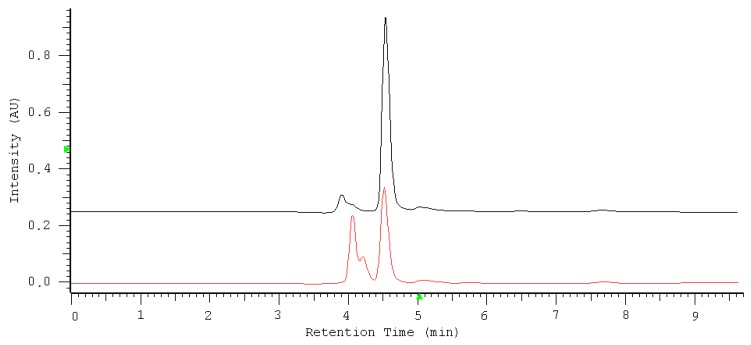
The overlapped chromatograms representing saliva with spiked nitrate and nitrite at a concentration level of 15 µg mL^−1^ without an oxidation reaction (red line) and saliva with spiked nitrate and nitrite after the oxidation reaction (black line).

The IAM.PC.DD2 Regis column showed sufficient affinity and selectivity for nitrate/nitrite and could be useful for the direct determination of these anions in saliva samples. Figure 7 shows overlapped chromatograms, one for the separation of the saliva sample before oxidation (red line) and the second after oxidation (blue line). The chromatograms show the specificity of the method towards nitrate undergoing quantification. The nitrate peak is well separated from all remaining sample components. To assess the selectivity and specificity of the method, the chromatograms of saliva with spiked nitrate and nitrite without an oxidation reaction (Figure 8A) and saliva with spiked nitrate and nitrite after the oxidation reaction (Figure 8B) are presented. The peaks were identified by comparing the retention time and absorption spectra of the sample with the standards. The agreement of the compared spectra was always higher than 0.9. The purity index measuring the spectral heterogeneity of a peak was always higher than 0.9, meeting the accepted requirements. 

The nitrite peak in the chromatogram is eluted quickly and is not well separated from other sample components. In this case, the use of the oxidation of nitrite to nitrate undergoing longer retention seems to be the best choice for their quantitative determination. After the oxidation step, the nitrite content is seen as the increase in the peak of nitrates. The quantification of nitrite/nitrate content in saliva samples is collected in Table 4. In addition, a peak of thiocyanate that disappears after oxidation has been identified. The product of the thiocyanate oxidation is the (CN)_2_ peak appearing at the beginning of the chromatogram:

10SCN^−^ + 14MnO_4_^−^ + 32 H_3_O^+^ ➞ 5(CN)_2_ + 10SO_4_^2−^ + 14Mn^2+^ + 48H_2_O

## 3. Materials and Methods

### 3.1. Materials and Sample

Deionized and purified water by ULTRAPURE Millipore Direct-Q 3UV-R (Merck, Darmstadt, Germany) with the resistivity 18.2 MΩ cm were used to prepare all the aqueous solutions. Sodium nitrate, sodium nitrite, sodium chloride, and potassium permanganate were sourced from POCH (Gliwice, Poland). Perchloric acid (70%, *w*/*v*) was purchased from Sigma-Aldrich (St. Louis, MO, USA). Stock standard solutions of sodium nitrate and sodium nitrite (100 mg·L^−1^) were prepared immediately before use.

### 3.2. HPLC Conditions

Chromatographic measurements were made on a LaChrom HPLC Merck Hitachi (E. Merck, Darmstadt, Germany) equipped with diode array detector (L-7455), pump (L-7100), interface (D-7000) and solvent degasser (L-7612). The chromatographic column IAM.PC.DD2 Regis HPLC (4.6 × 150 mm, 10 µm, pore size: 300 Å) was purchased from Agilent Technologies (Santa Clara, CA, USA). The column temperature was controlled in the range of 15–50 °C by the thermostatic column compartment L-7350. Chromatographic data were acquired and processed by D-7000 HSM Software version 3.0 (E. Merck, Darmstadt, Germany). The mobile phases were filtered through a Nylon 66 membrane filter (0.45 µm) Whatman (Maidstone, England) using a filtration apparatus. Aqueous NaCl was used as the mobile phase. The effect of NaCl concentration was studied in the range of 1–30 mM in water. Retention data was recorded at a flow-rate of 0.5 mL min^−1^ at room temperature (21 °C). The detection wavelength was set at 210 nm, chosen according to the recorded spectra by the DAD detector in the range of 190–400 nm. Ten microliters of the sample solution were injected three times. 

### 3.3. Method Validation

The analytical method validation was carried out according to the ICH Q2 (R1) method-validation guidelines [35]. The following validation parameters were established: selectivity, precision, accuracy, linearity, recovery, limit of detection (LOD), and limit of quantification (LOQ). 

#### 3.3.1. Linearity, Recovery, and Precision

The stock standard solutions of both salts were 100 mg L^−1^ in water. The working standard solution was prepared immediately before use. The serial dilutions were made to produce final concentrations in the range of 0.5–35.0 for nitrates and 0.5–15.0 mg L^−1^ for nitrites. The calibration curves for each analyte were obtained by injecting the sample into the HPLC and used as referential calibration curves for their quantification. Peak heights were measured for preparing calibration curves by employing least squares regression analysis. LOQ and LOD values were based on a 10:1 and 3:1 signal-to-noise ratio, respectively. 

#### 3.3.2. Recovery and Precision

The recovery study was accomplished by adding the reference standards to the saliva samples. Spiked samples without oxidation were prepared as follows: 1 mL of the standard solution, at different concentrations, was added to 1 mL of the fresh saliva. The oxidized spiked samples were prepared by mixing 1 mL of the spiked saliva with 10 µL of perchloric acid and 430 µL of 0.02 M KMnO_4_ and then made up to 2 mL with deionized water. Blank samples without oxidation were prepared by adding 1 mL of water to 1 mL of the fresh saliva. Blank oxidized samples were prepared by mixing 1 mL of the fresh saliva with 10 µL of HClO_4_ and 430 µL of 0.02 M KMnO_4_ and then made up to 2 mL with deionized water. The samples were heated at 100 °C for 15 min in a water bath MLL 147/ (AJL Electronic, Cracow, Poland) and then centrifuged at 9000× *g* for 45 min using the laboratory centrifuge MPW-223e (MPW Med. Instruments, Warsaw, Poland). Ten microliters of the clear supernatant were injected into the HPLC column. The analysis was repeated six times giving intraday precision values and six times on another day giving intermediate precision values. Inter-assay and intra assay variability were determined by computing the percentage relative standard deviation (%RSD). 

### 3.4. Saliva Sample Preparation

The saliva samples (*n* = 6) from healthy volunteers were analyzed *ex tempore* immediately after donation. The samples were prepared as follows: 1 mL of the fresh saliva was mixed with 1 mL of water (sample without oxidation step). The oxidized sample was prepared as follows: 1 mL of the fresh saliva was mixed with 10 µL of perchloric acid and 430 µL of 0.02 M KMnO_4_ and then made up to 2 mL with deionized water. After that, the mixtures were heated at 100 °C for 15 min, and then centrifuged at 9000× *g* for 45 min. A volume of the clear supernatant (10 µL) was injected into the HPLC system.

### 3.5. Data Analysis

Multiple regression analysis was performed by using Microsoft Excel 2010. Experimental values were expressed as the mean ± standard deviation (SD). R Core Team 2018 [36] software was used for statistical analyses, and the differences between groups were analyzed by one-way ANOVA test. The results were considered as statistically significant when *p* < 0.05.

## 4. Conclusion

It has been proven that phosphatidylcholines (PCs), owing to their interaction with inorganic anions, are able to modify the functioning of membrane processes [4]. In the current research, a chromatographic system consisting of an IAM.PC.DD2 Regis stationary phase formed by covalently bonding the phospholipids to a silica matrix was used to analyze chosen UV-absorbing anions, nitrate and nitrite, in saliva samples. The procedure was based on the oxidation reaction of nitrites to nitrates in acidic conditions by the use of potassium permanganate. Experimental conditions were optimized according to amount of mixture components and HPLC requirements. The kinetics of the permanganate–nitrite reaction was also measured chromatographically for the first time. Although the presence of nitrates and nitrites have been extensively investigated, especially in food, the phosphatidylcholine column has not been applied for this purpose so far. This prompted us to carry out the current research, in which we quantified nitrites after their oxidation to nitrates for the first time. We applied our method for analyte detection in saliva samples. Comparing the current methodology with that previously reported in the literature [37], the HPLC method with a UV-Vis detector and the sorbent RP18 with an ion-pair mode, utilizing octylamine as an ion-pair agent, regarding validation parameters, the phosphatidylcholine column ensured a roughly three-fold shorter retention time (current method: about 6 min, reported method: 15 min), and 100-fold smaller LOD value (current method: 4.56 and 4.21 µg L^−1^ for nitrates and nitrites, respectively, reported method: 25 and 50 mg L^−1^). In conclusion, the presented results prove the applicability of the IAM.PC.DD2 Regis HPLC column for nitrate and nitrite determination in saliva samples.

## Figures and Tables

**Table 1:** The linear regression parameters obtained for the calibration curves of nitrates and nitrites.

Linear Regression Parameters		Nitrates NO_3_^−^	Nitrites NO_2_^−^
LOD * [ng mL^−1^]		4.56	4.21
LOQ * [ng mL^−1^]		15.21	14.03
Linear range ** [µg mL^−1^]		0.50–35.00	0.50–15.00
Regression equation ***(y = ax + b) ****	a ± s_a_	7809.31 ± 97.92	8966.61 ± 194.40
	b ± s_b_	1739.12 ± 5114.79	−1743.60 ± 9500.16
	R^2^s_e_F	0.9992 6360.71 8703.09	0.9972 2127.55 18575.24

* Calculation from the signal-to-noise ratio (LOD: limit of detection and LOQ: limit of quantification, corresponding to 3 and 10 times the noise level, respectively); ** number of calibration points: 8; *** s_a_: the standard deviation of the slope (a), s_b_: the standard deviation of the intercept (b), s_e_: the standard error of the estimate, F-Fisher F statistic; **** x-concentration, y-peak height (nitrate) or y-increase of nitrate peak height after oxidation (nitrite).

**Table 2:** Recovery results. RSD: relative standard deviation.

**Parameters**	**Nitrate**		
Fortification level (µg/mL)	3.00	7.50	15.00
Average recovery (*n* = 6) (%)	98.12	98.65	93.33
Intra-day precision, (RSDr,%)	3.21	2.65	3.41
Inter-day precision, (RSDwR,%)	4.73	4.71	4.20
**Parameters**	**Nitrite**		
Fortification level (ng/mL)	3.00	7.50	15.00
Average recovery (*n* = 6) (%)	86.90	87.74	104.23
Intra-day precision, (RSDr,%)	3.51	3.83	3.34
Inter-day precision, (RSDwR,%)	5.90	4.19	4.76

RSDr: *Relative Standard Deviation* for repeatability, RSDwR: *Relative Standard Deviation* for reproducibility.

**Table 3:** Stability of the quantitation of nitrate in the water system and saliva samples (*n* = 6).

**Water System**			3 µg mL^−1^	7.5 µg mL^−1^	15 µg mL^−1^
	Without oxidation	tr	4.54 (±0.03)	4.58 (±0.05)	4.52 (±0.01)
		H	23604 (±342)	58198 (±989)	115198 (±1727)
		*A_s_*	1.15 (±0.02)	1.29 (±0.03)	1.28 (±0.02)
		*N*	5591 (±331)	5771 (±295)	5859 (±307)
		P	0.9996 (±0.0003)	0.9996 (±0.0001)	1.0000 (±0.0000)
		R^2^	0.9907 (±0.0014)	0.9907 (±0.0009)	0.9910 (±0.0003)
	After oxidation	tr	4.57 (±0.05)	4.53 (±0.03)	4.53 (±0.02)
		H	59305 (±771)	136869 (±1916)	262669 (±3415)
		*A_s_*	1.26 (±0.03)	1.34 (±0.04)	1.33 (±0.04)
		*N*	6122 (±262)	5960 (±311)	5944 (±270)
		P	1.0000 (±0.0000)	0.9996 (±0.0002)	0.9999 (±0.0001)
		R^2^	0.9908 (±0.0011)	0.9910 (±0.0007)	0.9908 (±0.0009)
**Samples of Saliva**	Without oxidation	tr	4.53 (±0.03)	4.55 (±0.04)	4.58 (±0.06)
		H	72097 (±901)	108175 (±1293)	167554 (±1508)
		*A_s_*	1.36 (±0.03)	1.28 (±0.02)	1.25 (±0.04)
		*N*	5446 (±369)	5365 (±291)	5850 (±283)
		P	0.9614 (±0.0097)	0.9731 (±0.0054)	0.9982 (±0.0016)
		R^2^	0.9909 (±0.0012)	0.9908 (±0.0008)	0.9907 (±0.0008)
	After oxidation	tr	4.51 (±0.02)	4.53 (±0.01)	4.55 (±0.04)
		H	123968 (±1759)	197560 (±2374)	340325 (±3544)
		*A_s_*	1.37 (±0.05)	1.22 (±0.02)	1.35 (±0.03)
		*N*	6499 (±321)	6036 (± 279)	6450 (±332)
		P	0.9997 (±0.0002)	0.9998 (±0.0002)	1.0000 (±0.0000)
		R^2^	0.9906 (±0.0005)	0.9904 (±0.0012)	0.9904 (±0.0007)

Abbreviations: tr—peak retention time, H—peak height, *A_s_*—asymmetry factor, *N*—theoretical plate number, P—peak purity, R^2^—the correlation coefficient for two compared spectra.

**Table 4:** Nitrate/nitrite levels in saliva samples (*n* = 6).

Nitrate		Nitrite	
Concentration Range [µg mL^−1^]	Mean ± SD [µg mL^−1^]	Concentration Range [µg mL^−1^]	Mean ± SD [µg mL^−1^]
8.98–18.52	13.76 ± 3.20	3.50–5.34	4.24 ± 0.65
